# Highly targeted cholera vaccination campaigns in urban setting are feasible: The experience in Kalemie, Democratic Republic of Congo

**DOI:** 10.1371/journal.pntd.0006369

**Published:** 2018-05-07

**Authors:** Louis Albert Massing, Soumah Aboubakar, Alexandre Blake, Anne-Laure Page, Sandra Cohuet, Adalbert Ngandwe, Eric Mukomena Sompwe, Romain Ramazani, Marcela Allheimen, Philippe Levaillant, Pauline Lechevalier, Marie Kashimi, Axelle de la Motte, Arielle Calmejane, Malika Bouhenia, Ernest Dabire, Didier Bompangue, Benoit Kebela, Klaudia Porten, Francisco Luquero

**Affiliations:** 1 Médecins Sans Frontières, Katanga, Democratic Republic of Congo; 2 Epicentre, Paris, France; 3 Ministère de la Santé Publique, Katanga, Democratic Republic of Congo; 4 Médecins Sans Frontières, Paris, France; 5 World Health Organization, Kinshasa, Democratic Republic of Congo; 6 Ministère de la Santé Publique, Kinshasa, Democratic Republic of Congo; International Vaccine Institute, REPUBLIC OF KOREA

## Abstract

**Introduction:**

Oral cholera vaccines are primarily recommended by the World Health Organization for cholera control in endemic countries. However, the number of cholera vaccines currently produced is very limited and examples of OCV use in endemic countries, and especially in urban settings, are scarce. A vaccination campaign was organized by Médecins Sans Frontières and the Ministry of Health in a highly endemic area in the Democratic Republic of Congo. This study aims to describe the vaccine coverage achieved with this highly targeted vaccination campaign and the acceptability among the vaccinated communities.

**Methods and findings:**

We performed a cross-sectional survey using random spatial sampling. The study population included individuals one year old and above, eligible for vaccination, and residing in the areas targeted for vaccination in the city of Kalemie. Data sources were household interviews with verification by vaccination card. In total 2,488 people were included in the survey. Overall, 81.9% (95%CI: 77.9–85.3) of the target population received at least one dose of vaccine. The vaccine coverage with two doses was 67.2% (95%CI: 61.9–72.0) among the target population. The vaccine coverage was higher during the first round (74.0, 95%CI: 69.3–78.3) than during the second round of vaccination (69.1%, 95%CI: 63.9–74.0). Vaccination coverage was lower in male adults. The main reason for non-vaccination was to be absent during the campaign. No severe adverse events were notified during the interviews.

**Conclusions:**

Cholera vaccination campaigns using highly targeted strategies are feasible in urban settings. High vaccination coverage can be obtained using door to door vaccination. However, alternative strategies should be considered to reach non-vaccinated populations like male adults and also in order to improve the efficiency of the interventions.

## Author summary

The oral cholera vaccine, Shanchol, has already been shown as an effective tool in controlling a cholera outbreak. The limited amount of doses, concurrently with the logistic constraints associated with a targeted vaccination campaign are serious difficulties to tackle in order to organize a vaccination campaign in an urban setting. Although the World Health Organization recommends its use for cholera control in endemic countries, the fact remains that the use of the oral cholera vaccine in endemic setting has scarcely been described, especially in an urban setting, until now. Médecins Sans Frontières and the Ministry of Health from Democratic Republic of Congo organized a vaccination campaign of a limited part of the urbanized and highly endemic city of Kalemie, in the Tanganyika Province using a door to door strategy. The vaccine coverage in the targeted zones was high and demonstrated the feasibility of cholera vaccination campaign in this setting but also the need for creative strategies in order to reach population remaining hard to vaccine.

## Introduction

Cholera is endemic [1] in several countries in Africa and Asia [2,3] and can also cause large national wide epidemics, often involving several countries [4,5]. The World Health Organization (WHO) with financial support from GAVI, the Vaccine Alliance, has established an oral cholera vaccine (OCV) stockpile for emergency response conceived to respond to outbreaks and humanitarian crises where the risk of cholera is high. In addition, the Global Task Force for Cholera Control manages a non-emergency reserve that is expected to be used in endemic countries or in outbreak and humanitarian crises if the emergency stockpile is insufficient to cover the demand. However, the number of cholera vaccines currently produced is very limited and examples of OCV use in endemic countries, and especially in urban settings, are scarce.

Kalemie, a city located in Tanganyika province in the Democratic Republic of Congo (DRC), is considered one of the urban hotspot in the National Cholera Control Plan of DRC and it is mentioned a priority area to carry out control interventions in this country. Médecins Sans Frontières (MSF) has provided support to the Ministry of Health (MoH) in Kalemie in different cholera control activities using an integrated approached as it is recommended in the National Cholera Control Plan. These activities include: treatment of cholera cases, diseases surveillance, improvements in access to safe water and health promotion. The most ambitious part of this intervention was the building of a new water supply network to bring water to the health areas of Undugu and Kituku where the access to safe water was poor and cholera is highly endemic. In addition, MSF and the MoH planned to organize an OCV vaccination campaign in 2013 as additional control measures, to cover the gap during the time that the main component of the intervention was implemented.

MSF requested a loan to the MSF requested a loan to the International Coordinating Group (ICG), that manages the global stockpile of oral cholera vaccines, and 252,000 doses with a close expiration date (3 months for 42% of the doses provided and 9 months for the rest) were approved for shipment to Kalemie. The vaccination campaign was planned to cover the four health areas with the highest historical attack rates, covering a population around 120,000 people. Both the MoH and the MSF feared that this highly targeted approach could generate tensions within the city, especially in neighborhoods not receiving the vaccine. Thus, the selected vaccination strategy was door to door, to avoid mass gathering, and the social mobilization campaign was light with message passed only within the targeted communities through community health workers without using mass media. The vaccination campaign started in November 25; however, the three days later, the team was evacuated for a complex security incident unrelated to the campaign. One month later (end of December 2013) the vaccination team was allowed to come back to Kalemie, however 41.6% of the initial doses achieved the expiration date and were discarded. The MSF team re-discussed with the MoH the vaccination strategy and the target population was reduced to two health areas (Undugu and Kataki), with the highest historical incidence rates, covering a population around 50,000 people. The initial vaccination strategy was maintained and the campaign started on July 1, 2014. Two doses, 14 days apart, were provided to all individuals one year and above excluding women who orally reported to be pregnant due to the concerns from local authorities and the lack of safety data for this group at the time.

Here we describe the vaccine coverage achieved with this highly targeted vaccination campaign and the acceptability of the campaign in Kalemie.

## Methods

### Study setting

**Fig 1:**
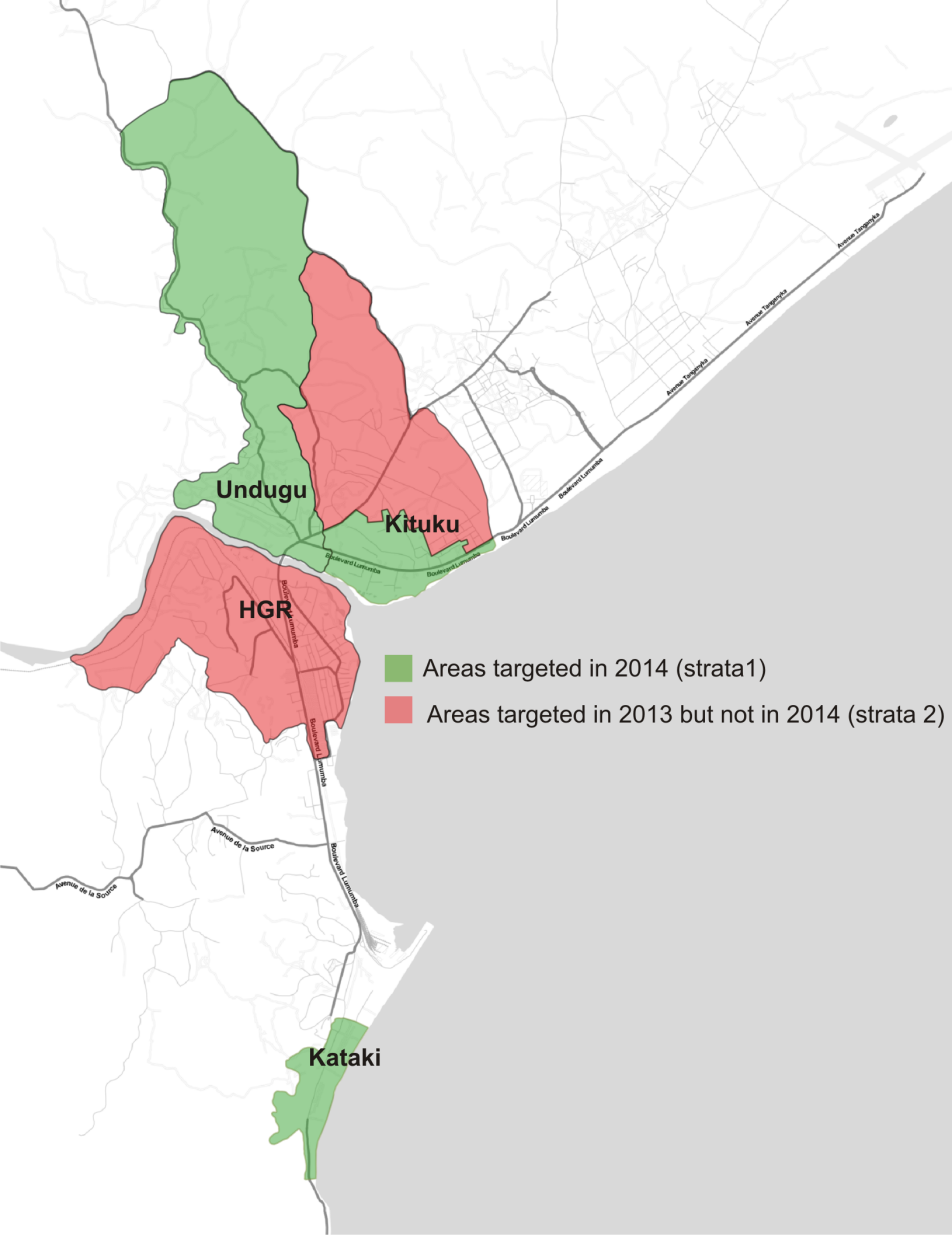
Vaccinated and non-vaccinated health areas of the city of Kalemie, Democratic Republic of Congo (base map: Stamen Design).

The city of Kalémie is located in Katanga, in Eastern Health District of Tanganyika in the DRC, near the lake of the same name. The Lukuga River separates the city into southern districts (eight health areas) and northern districts (three health areas) (Fig 1). The population of Kalémie is estimated at 262,963 people. Cholera cases are reported throughout the year in Kalémie with two seasonal peaks, one in September-October and another in January. All cases of cholera are treated in the diarrheal diseases treatment center of the General Referral Hospital of Kalémie. The health areas reporting the highest attack rates are located along the Lukuga River and Lake Tanganyika (Kituku Undugu in Nyemba; HGR and Kataki) (Fig 1).

### Study design and participants

We carried out a cross-sectional survey in the areas included as part of the target population of the 2014 OCV campaign (strata 1) in Kalemie including Undugu, Kataki and three neighborhoods of Kituku (Tanganika, Quartier Industriel and Central-Singa). We also assess the vaccination coverage of the three days of vaccine distribution in November, 2013 in HGR and the neighborhoods of Kituku not included in 2014 (strata 2).

All individuals residing in the health areas targeted for vaccination were eligible for inclusion in the survey (Fig 1). Residents were defined as persons living (sleeping and eating) in the area since the starting date of the vaccination campaign. Households were selected using spatial random sampling [6]. A household was defined as a group of people sleeping under the same roof and sharing meals every day for at least the previous two weeks to the starting date of the vaccination campaign.

### Sample size

The sample size was calculated to obtain a representative estimate of the proportion of residents who received two doses of OCV by age group (1–4, 5–14, 15 years and older) in the vaccinated areas in 2014 (strata 1). Sample size was calculated to ensure a sufficiently precise estimate for children aged 1 to 4 years as this group was the smallest. We considered the following assumptions: 85% of children would receive two doses of vaccine, alpha error of 5%, and absolute precision of 5%. Taking into account a census conducted prior the campaign in October 2013, we expected 0.9 children 1–4 year old per household (average of 6.2 individuals per household and 14% of the population between 1 and 4 years). Assuming 10% of missing data, we planned to visit 202 households and include all household members.

For the non-vaccinated areas in 2014 that were initially targeted in 2013 (strata 2) it was difficult to forecast the coverage, however we expected a two dose coverage lower than 15% in this population, thus the same sample size was considered in this second strata.

### Data collection

Teams conducted face-to-face directed interviews after consent. A standardized pre-piloted questionnaire was used to collect the following information: demographic data (age, sex, and household size), vaccination status (card-confirmed and orally reported), reasons for non-vaccination (open question), and acceptability data (adverse events, taste and beliefs about the vaccine). Questions concerning acceptability were only collected in participants older than 15 years. Interviews were conducted in the local language, Swahili.

Survey teams asked for the help of neighbors to trace absentees and re-visit empty (but not abandoned) households later in the day. If during the second visit the occupants could not be found or if they refused to participate, that household was skipped.

### Data entry and analysis

Our main outcome was the OCV coverage (full course and at least one dose) in the target population. Vaccine coverage was calculated dividing the number of individuals reporting being vaccinated by the survey population and expressed as a percentage. Vaccination coverage estimates include both card-confirmed and oral reporting. Secondary outcomes included vaccine coverage by age group, sex, and reasons for non-vaccination. We also estimated the geographical distribution of the vaccine coverage using spline functions [7]. Crude vaccination coverage estimates and 95% confidence intervals (95% CI) were obtained considering the survey design. The design effect (deff) was calculated to estimate the loss of precision due to the selection of all member of the household.

The geographical distribution of the vaccine coverage was estimated using a binomial regression in a general additive model framework. The dependent variable was the vaccination status of each individual, and the location of the household was included as a smoothing spline term. The smoothing parameter of the spline term was chosen by cross validation. We plot the vaccine coverage and the standard deviation of the term as an indicator of the uncertainty in the estimates.

The reasons for non-vaccination were entered in French by data entry clerks speaking fluently French and Swahili. Key words associated with different categories of answer were identified. An automatic detection of those key words allowed to categorize the answers.

Data entry was performed using EpiData 3.1 (EpiData Association, Denmark) and data analysis was performed using Stata 13.0 (College Station, USA) and R Statistical Software (The R Foundation for Statistical Computing).

### Ethical considerations

The Ethical Review Board of the University of Lubumbashi approved the study protocol (study protocol ethical number: UNILU/CEM/028/2013). Oral informed consent was obtained from participants in all instances. All children had consent given from a parent/guardian and all adult participants provided their own consent. Oral informed consent was requested since the study did not present any risk of harm to subjects and did not involve procedures for which written consent is normally required outside the research context. The request of consent was registered in a log-book. Privacy and confidentiality of the data collected from participants were ensured both during and after the conduct of the surveys.

## Results

### Description of the population

Overall, 398 households were visited between August 21 and 29, 2014 and 2,465 individuals were included as part of the survey. Among these individuals 48 were excluded in the analysis; 8 were children less than 1 year of age and 40 were pregnant women. Finally, 2,417 were included; 1,160 in the first strata (areas targeted in the 2014 campaign) and 1,257 in second strata (initially included in the 2013 campaign but not targeted in 2014). The average household size was 6.4 (standard deviation: 3.2). The median age was 15 year old (inter quantile range (IQR): 7–28 years). The male:female ratio was 0.92 (95%CI: 0.84–0.99).

### Vaccine coverage

#### Strata 1: Vaccinated areas in 2014: Undugu, HGR and part of Kituku

Overall 81.9% (n = 950/1160; 95%CI: 77.9–85.3, deff = 2.8) of the target population received at least one dose of vaccine. The vaccine coverage with two doses was 67.2% (n = 779/1160; 95%CI: 61.9–72.0, deff = 3.5) among the target population. The vaccine coverage was higher during the first round (74.1% 95%CI: 69.3–78.3, deff = 3.2) than during the second round of vaccination (69.1% 95%CI: 63.9–73.9, deff = 3.6). In the mop-up campaign organized after the second round, 5.9% (95%CI: 3.6–9.5, deff = 4.6) of the population was vaccinated. The dropout rate between the first and second round was 15.3% (95%CI: 13.0–17.8, deff = 3.8). The percentage of individuals showing the vaccination card among those reporting being vaccinated was 66.2% (95%CI: 58.8–72.9, deff = 4.9) in the first round, 63.6% (95%CI: 55.8–70.7, deff = 5.0) in the second round and 58.8% (95%CI: 30.9–82.0, deff = 5.3) in the third round.

**Fig 2:**
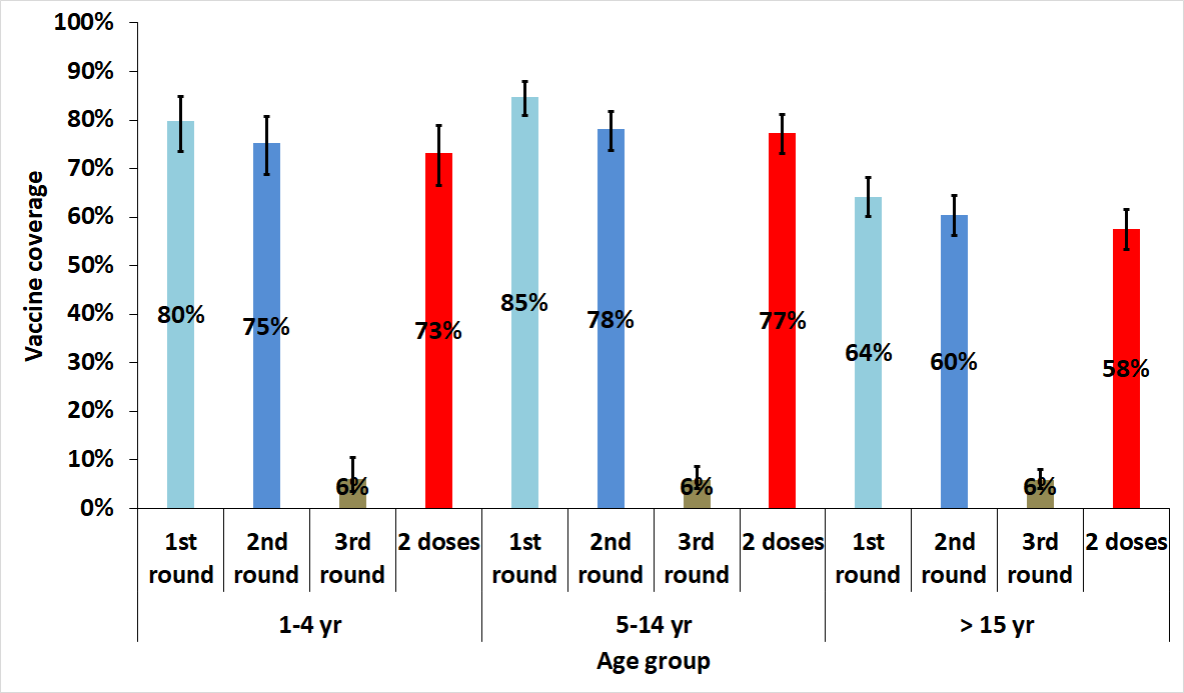
Vaccination coverage by age in the areas targeted for vaccination in 2014.

The vaccination coverage was higher among children 5 to 14 years old than in adults and small children (Fig 2). The vaccination coverage was higher among women 85.7% (95%CI: 81.1–89.4, deff = 2.2) than in among men 77.5% (95%CI: 72.8–81.5, deff = 1.5) in all age groups.

#### Strata 2: Non vaccinated areas in 2014 that were targeted in 2013: HGR and parts of Kituku

Overall, 31.7% (n = 398/1257; 95%CI: 26.0–37.9, deff = 5.3) of the population in the strata 2 received at least one dose of vaccine; 86.9% (95%CI: 76.1–93.3) received one in 2013 and 16.3% (95%CI: 9.1–27.6) in 2014. The vaccine coverage with two doses was 3.3% (n = 41; 95%CI: 1.4–7.2, deff = 7.2) among those living in strata 2. The percentage of individuals showing the vaccination card among those reporting being vaccinated was 96.9% for the campaign in 2014, 34.6% for the campaign in 2013.

### Geographical distribution of the vaccination coverage

**Fig 3:**
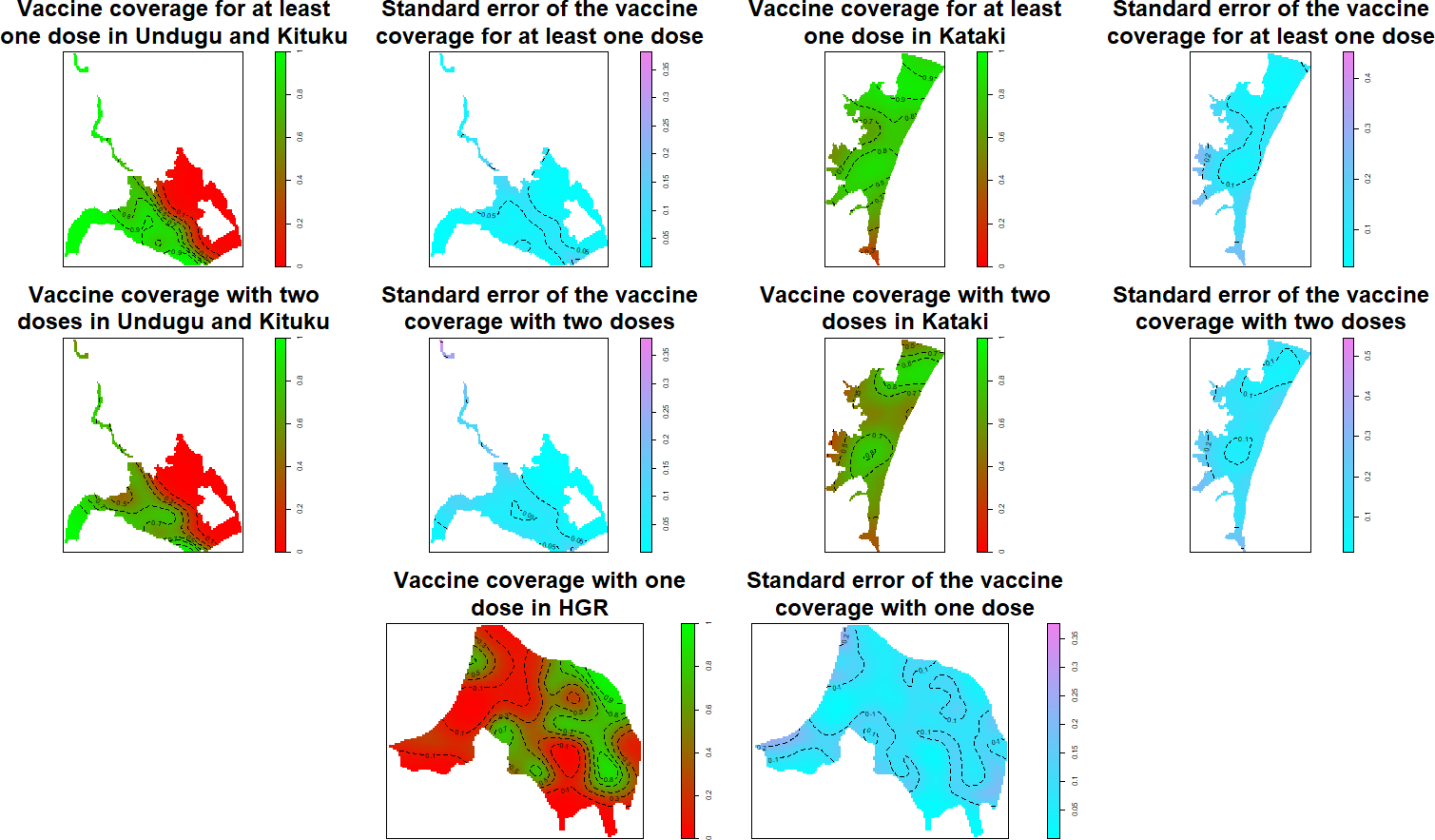
Spatial distribution of the vaccination coverage in Kalemie.

Vaccination coverage varied within the areas targeted in these campaigns. In the three areas covered by the campaign in 2014 (Undugu, Katataki and the three neighborhoods of Kituku) we observed areas with coverage of two doses close to 100% and areas with coverage as low as 30% (Fig 3). The uncertainty about the estimation was relatively low as shown in Fig 3. The coverage in strata 2 was as well heterogeneous with a high percentage of individuals vaccinated with at least one dose in HGR (Fig 3).

### Reasons for non-vaccination in the 2014 campaign (strata 1)

The main reason for non-vaccination was obtained for all 1301 and 358 people who had not received vaccine during the first and second round of vaccination in 2014, respectively, and for 368 people who had received zero (n = 204) or one dose (n = 164) during the first two rounds in 2014 and had not received the dose during the mop-up campaign (Table 1). The main reason for non-vaccination was to be absent during the campaign, representing the main reason for more than 2/3 of the individuals. Between 5% and 7% of non-vaccinated refused the vaccine, either for religious reasons or tradition, or because they considered that the vaccine was unsafe. The proportion of persons who have not been vaccinated in 2014 for lack of information was very low (Table 1).

**Table 1:** Reasons for non-vaccination in the 2014 campaign (strata 1).

	1st round	2nd round	3rd round
	N = 301	N = 358	N = 368
The participant was absent the day of the vaccine distribution	244 (81.1)	254 (70.9)	256 (69.6)
Refusal linked with cultural or religious beliefs	12 (4.0)	12 (3.4)	10 (2.7)
The vaccine was considered dangerous	9 (3.0)	9 (2.5)	9 (2.5)
The participant had an adverse effect after the first dose	0 (0)	9 (2.5)	9 (2.5)
The participant was ill during the vaccination campaign	5 (1.7)	6 (1.7)	6 (1.6)
The participant did not know about the vaccination campaign	3 (1.0)	5 (1.4)	15 (4.1)
Bad previous experience with other vaccines	3 (1.0)	4 (1.1)	4 (1.1)
The participant did not have time for the vaccination	1 (0.3)	7 (2.0)	7 (1.9)
The vaccinator decided not to vaccinate the participant	1 (0.3)	3 (0.8)	1 (0.3)
The participant did not know the date of the campaign	1 (0.3)	1 (0.3)	1 (0.3)
No explanation	8 (2.7)	22 (6.1)	22 (6.0)
Other	14 (4.0)	26 (7.0)	28 (6.5)

### Adverse events following immunization in the 2014 campaign (strata 1)

During the vaccine coverage survey, a part of the interview focused on the adverse event following immunization (first and second round of 2014). Among those who received the vaccine, 4.3% (n = 39) reported having been ill after the first round of vaccination and 1.4% (n = 13) after the second round. The main signs or symptoms reported over the two rounds were diarrhea (22.4%), fever (31.0%), stomachache (15.5%) nausea and vomiting (12.0%). Among those who said they experienced side effects, 36.4% (n = 12/33) reported consulting after the first round, 30.8% (n = 4/13) after the second round.

### Knowledge and acceptability of the 2014 vaccination campaign (strata 1)

Among the 367 participants aged at least 15 years included in the survey in the strata 1 (targeted areas in the 2014 campaign), 334 reported being aware of the vaccination campaign in 2014 (91.0%, 95% CI: 87.0 to 93.9). Around half of them thought that the vaccine had a good effect on their health status (51.0%, 95% CI: 43.4 to 58.5), while a quarter (26.4%, 95% CI: 20.6 to 33.2) felt that the vaccine had no effect and 8.2% (95% CI 4.9 to 13.3) a bad effect. In addition, 15.5% (95% CI: 10.2 to 23.0) thought that the vaccine could cause illness. Most of those participants vaccinated reported that the vaccine had a bad taste (85.1%, 95% CI: 79.7 to 89.3). However, the vast majority of respondents said they would go to get vaccinated again if a new vaccination campaign was organized in the future (93.4%, 95% CI: 88.9 to 96.2).

## Discussion

This study shows that highly targeted mass vaccination campaigns in urban settings are feasible and that high vaccine coverage can be reached within the areas selected for vaccination. The campaign was organizing without major logistic difficulties and any civil unrest. Most of the doses were distributed in the predefined target areas for vaccination; however, some spillover of vaccine in the surrounding areas was documented. This issue has been already reported even in rural settings, where it is easier to target specific villages [8]. This is one of the first times that OCV has been used in urban setting outside a vaccine trial. The only other example of highly targeted vaccination in an urban African setting was a demonstration project carried out in Beira, Mozambique, where a lower coverage was achieved [9]. In that case, the strategy proved as well to be feasible and well accepted by the local community [9]. Feasibility and acceptability of this highly targeted approach in urban settings is similar to what has been described for rural areas or refugee camp settings [10,11].

Interestingly, the data from the coverage survey did not correlate well with the administrative coverage. The administrative coverage was close to 100% since all the vaccines were use and the number of vaccine doses available was close to the target population for the 2014 campaign. This means that some of the vaccines that should have been delivered in the strata 1 were provided to people not living in that area or who were not initially part of the estimated target population. This could be explained as a combination of two different issues: (i) individuals living in bordering areas came to be vaccinated into the strata 1, (ii) there was an increase in the target population between the census in October 2013 and the implementation of the camping in July 2014. We have indications that probably the two processes occurred in Kalemie. The coverage survey revealed that some people were vaccinated in strata 2 during the 2014 campaign even in they were not part of the target population. In addition the administrative coverage was over 100% in children under five which could be the result of children from bordering areas coming to get vaccine. This could be partially also the result of a high fertility rate in those communities that end up increasing the denominator among children 1 to 5 years old. This hypothesis is supported by a slight reduction in the age average in the survey sample compared with the census. Additionally, the survey showed a slightly higher average household size than the census, which supports as well the increased size of the target population linked with the high fertility rate.

All the doses available were distributed in 2014 but the data shows that especially male adults were vaccinated at lower rates that the rest of the population. A small mop-up activity was needed to complete this distribution of the doses, but was clearly insufficient to reach all the unvaccinated individuals. One of the reasons that might explain the lower coverage among adults is the vaccination strategy itself. The door to door distribution benefited the intake among those who tend to spend more time at home. In addition, the social mobilization activities was implemented with a low profile because of the fear of running out of vaccine doses if the people from surrounding areas would have come massively to get vaccination. These two issues highlight the need to find innovative strategies to reach population, like the male adults, that systematically show lower vaccine coverage in cholera vaccination campaigns [8,12–14]. The lesser coverage of adult men could be due to lower availability because of work during daytime or because of the perception that the vaccine is mainly targeting children as it has already been suggested in other vaccination campaigns [8,15]. A possible solution might be to change the unit for vaccine distribution from the individual to the household, having a senior adult responsible of delivering the vaccine to the entire family such as the self-administration strategy adopted to distribute the second dose to fishermen in Malawi [15]. This could reduce the cost and increase the efficiency of the campaigns. Alternative vaccination strategies should be design taking into account three characteristics of the vaccine that can increase the field effectiveness: the efficacy of the first dose [16], the high thermal-stability of the lipopolysaccharide (main antigen of Shanchol) [17], and the high indirect protection provided when high vaccine coverage is achieved [18–20].

Related to this last point, an element to consider when designing future campaigns should be the geographical heterogeneity, which has been previously described in other campaigns [12]. A large part of the potential of cholera vaccination campaigns to reduce cases and deaths is expected to be obtained through to the indirect protection induced by this vaccine [21,22]. Therefore, the existence of pockets of non-vaccinated individuals represents a risk of cholera spread in cholera prone areas. Real-time information on the distribution of the vaccination coverage could help to reduce these geographical disparities, which represents one of the major risks than could reduce the impact of oral cholera vaccines. In addition, in absence of enough vaccine to cover the desire vaccine coverage, as it was the case in Kalemie, modelling work suggest that might be more beneficial in the short term to provide one dose to a larger population than two doses to a smaller number of individuals [22].

The major limitation in our evaluation was the high percentage of vaccinated individuals with missing vaccination cards despite the short interval between the vaccination camping and the coverage survey. We think that this short delay limits the risk of having a large information bias in the vaccination status; however, it highlights the difficulty to be certain about the vaccination status, which might be the major limitation to understand the effectiveness and impact of vaccination campaigns in the medium-long term.

The sample size that we expected for each stratum was achieved, and this allowed having adequate precision in the estimates, despite the slightly lower vaccine coverage than the one assumed in the sample size calculation. In strata 2, where we had a higher uncertainty about coverage, the two dose coverage was lower than the 15% assumed in the sample size calculation and thus we had a good overall precision in our estimates.

An additional limitation is the use the vaccine coverage and other quantitative measure to evaluate the acceptability of the campaign. Qualitative assessment could enrich the knowledge that we have about the knowledge, perceptions and barriers that limits the performance of the vaccination activities. Qualitative measurements are needed to understand the best approach to vaccinate mobile communities like the fishermen in Kataki area at it has been shown in refugee camps settings like South Sudan [23].

Despite the robust sampling method, the potential lower availability of adult men during daytime, as reflected by our sex ratio slightly but significantly lower than 1, could have resulted in a slight overestimate of the global vaccine coverage, since the coverage appeared significantly lower in this group. However a sex ratio lower than 1 could also simply reflect a higher mortality rate of adult male, as is frequent in African settings.

In conclusion, the vaccination campaign in Kalemie was implemented using a highly targeted vaccination strategy without major logistical difficulties. The vaccination coverage obtained was relatively high, but lower than expected, especially among the male adults. The relative low coverage among male adults highlights the need to find better vaccination strategies and to consider alternative methods for vaccine distribution for specific subpopulations. These alternative vaccination protocols should take full advantage of the relative high vaccine efficacy of the first vaccine dose and the good thermal-stability of the vaccine.

## Supporting information

S1 Checklist STROBE checklist. (DOCX)
