# Supplementary material for: Highly targeted cholera vaccination campaigns in urban setting are feasible: The experience in Kalemie, Democratic Republic of Congo
Source: PLoS Negl Trop Dis. 2018 May 7;12(5):e0006369. doi: 10.1371/journal.pntd.0006369 (PMC5957443; doi:10.1371/journal.pntd.0006369)
Supplement: S1 Checklist — (DOCX) [file pntd.0006369.s001.docx]

STROBE Statement—checklist of items that should be included in reports of observational studies

|  | Item No. | Recommendation | Page  No. | Relevant text from manuscript |
| --- | --- | --- | --- | --- |
| **Title and abstract** | 1 | (*a*) Indicate the study’s design with a commonly used term in the title or the abstract | Page 2 | Abstract: Methods and Findings |
|  |  | (*b*) Provide in the abstract an informative and balanced summary of what was done and what was found | Page 2 | Abstract: Methods and Findings |
| Introduction | | | |  |
| Background/rationale | 2 | Explain the scientific background and rationale for the investigation being reported | Page 5 | Introduction: paragraph 1 |
| Objectives | 3 | State specific objectives, including any prespecified hypotheses | Page 6 | Introduction: paragraph 4 |
| Methods | | | |  |
| Study design | 4 | Present key elements of study design early in the paper | Page 7 | Methods: Study design and participants: paragraph 1 |
| Setting | 5 | Describe the setting, locations, and relevant dates, including periods of recruitment, exposure, follow-up, and data collection | Page 7  Page 8 | Methods: Study setting: paragraph 1  Methods: Data collection: paragraph 1 |
| Participants | 6 | (*a*) *Cohort study*—Give the eligibility criteria, and the sources and methods of selection of participants. Describe methods of follow-up  *Case-control study*—Give the eligibility criteria, and the sources and methods of case ascertainment and control selection. Give the rationale for the choice of cases and controls  *Cross-sectional study*—Give the eligibility criteria, and the sources and methods of selection of participants | Page 7 | Methods: Study design and participants: paragraph 2 |
|  |  | (*b*) *Cohort study*—For matched studies, give matching criteria and number of exposed and unexposed  *Case-control study*—For matched studies, give matching criteria and the number of controls per case |  |  |
| Variables | 7 | Clearly define all outcomes, exposures, predictors, potential confounders, and effect modifiers. Give diagnostic criteria, if applicable | Page 8 | Methods: Data collection: paragraph 1 |
| Data sources/ measurement | 8* | For each variable of interest, give sources of data and details of methods of assessment (measurement). Describe comparability of assessment methods if there is more than one group | Page 8 | Methods: Data collection: paragraph 1 |
| Bias | 9 | Describe any efforts to address potential sources of bias |  |  |
| Study size | 10 | Explain how the study size was arrived at | Page 8 | Methods: Sample size: paragraphs 1 and 2 |

Continued on next page

| Quantitative variables | 11 | Explain how quantitative variables were handled in the analyses. If applicable, describe which groupings were chosen and why | Page 9 | Methods: Data Entry and Analysis: paragraphs 1 and 2 |
| --- | --- | --- | --- | --- |
| Statistical methods | 12 | (*a*) Describe all statistical methods, including those used to control for confounding | Page 9 | Methods: Data Entry and Analysis: paragraphs 1 and 2 |
|  |  | (*b*) Describe any methods used to examine subgroups and interactions |  |  |
|  |  | (*c*) Explain how missing data were addressed |  |  |
|  |  | (*d*) *Cohort study*—If applicable, explain how loss to follow-up was addressed  *Case-control study*—If applicable, explain how matching of cases and controls was addressed  *Cross-sectional study*—If applicable, describe analytical methods taking account of sampling strategy | Page 9 | Methods: Data Entry and Analysis: paragraphs 1 and 2 |
|  |  | (*e*) Describe any sensitivity analyses |  |  |
| Results | | | | |
| Participants | 13* | (a) Report numbers of individuals at each stage of study—eg numbers potentially eligible, examined for eligibility, confirmed eligible, included in the study, completing follow-up, and analysed | Page 11 | Results: Description of the population: paragraph 1 |
|  |  | (b) Give reasons for non-participation at each stage | Page 11 | Results: Description of the population: paragraph 1 |
|  |  | (c) Consider use of a flow diagram |  |  |
| Descriptive data | 14* | (a) Give characteristics of study participants (eg demographic, clinical, social) and information on exposures and potential confounders | Page 11 | Results: Description of the population: paragraph 1 |
|  |  | (b) Indicate number of participants with missing data for each variable of interest |  |  |
|  |  | (c) *Cohort study*—Summarise follow-up time (eg, average and total amount) |  |  |
| Outcome data | 15* | *Cohort study*—Report numbers of outcome events or summary measures over time |  |  |
|  |  | *Case-control study—*Report numbers in each exposure category, or summary measures of exposure |  |  |
|  |  | *Cross-sectional study—*Report numbers of outcome events or summary measures | Pages 11 and 12 | Results: Vaccine coverage: Strata 1: Vaccinated areas in 2014: Undugu, HGR and part of Kituku  Results: Vaccine coverage: Strata 2: Non vaccinated areas in 2014 that were targeted in 2013: HGR and parts of Kituku |
| Main results | 16 | (*a*) Give unadjusted estimates and, if applicable, confounder-adjusted estimates and their precision (eg, 95% confidence interval). Make clear which confounders were adjusted for and why they were included | Pages 11 and 12 | Results: Vaccine coverage: Strata 1: Vaccinated areas in 2014: Undugu, HGR and part of Kituku  Results: Vaccine coverage: Strata 2: Non vaccinated areas in 2014 that were targeted in 2013: HGR and parts of Kituku |
|  |  | (*b*) Report category boundaries when continuous variables were categorized |  |  |
|  |  | (*c*) If relevant, consider translating estimates of relative risk into absolute risk for a meaningful time period |  |  |

Continued on next page

| Other analyses | 17 | Report other analyses done—eg analyses of subgroups and interactions, and sensitivity analyses | Page 12  Page 13  Page 14 | Results: Geographical distribution of the vaccination coverage  Results: Reasons for non-vaccination in the 2014 campaign  Results: Adverse-events following immunization in the 2014 campaign  Results: Knowledge and acceptability of the 2014 vaccination campaign |
| --- | --- | --- | --- | --- |
| Discussion | | | | |
| Key results | 18 | Summarise key results with reference to study objectives | Page 15 | Discussion: paragraph 1 |
| Limitations | 19 | Discuss limitations of the study, taking into account sources of potential bias or imprecision. Discuss both direction and magnitude of any potential bias | Page 17 | Discussion: paragraphs 5 and 7 |
| Interpretation | 20 | Give a cautious overall interpretation of results considering objectives, limitations, multiplicity of analyses, results from similar studies, and other relevant evidence | Page 17 | Discussion: paragraph 8 |
| Generalisability | 21 | Discuss the generalisability (external validity) of the study results | Page 15  Page 16 | Discussion: paragraph 1  Discussion: paragraph 3 |
| Other information | |  | | |
| Funding | 22 | Give the source of funding and the role of the funders for the present study and, if applicable, for the original study on which the present article is based |  |  |

*Give information separately for cases and controls in case-control studies and, if applicable, for exposed and unexposed groups in cohort and cross-sectional studies.

**Note:** An Explanation and Elaboration article discusses each checklist item and gives methodological background and published examples of transparent reporting. The STROBE checklist is best used in conjunction with this article (freely available on the Web sites of PLoS Medicine at http://www.plosmedicine.org/, Annals of Internal Medicine at http://www.annals.org/, and Epidemiology at http://www.epidem.com/). Information on the STROBE Initiative is available at www.strobe-statement.org.
